# Comprehensive pan-cancer analysis of N7-methylguanosine regulators: Expression features and potential implications in prognosis and immunotherapy

**DOI:** 10.3389/fgene.2022.1016797

**Published:** 2022-10-21

**Authors:** Wei Wei, Chao Liu, Caihong Wang, Meng Wang, Wei Jiang, Yaqian Zhou, Shuqun Zhang

**Affiliations:** ^1^ Department of Oncology, The Second Affiliated Hospital of Xi’an Jiaotong University, Xi’an, China; ^2^ Department of Vascular Surgery, The First Affiliated Hospital of Xi’an Jiaotong University, Xi’an, China; ^3^ Department of Pathology, The Second Affiliated Hospital of Xi’an Jiaotong University, Xi’an, China; ^4^ College of Chemistry and Materials Science, Northwest University, Xi’an, Shaanxi, China

**Keywords:** N7-methylguanosine, immunotherapy, prognosis, tumor immune microenvironment, pan-cancer analysis

## Abstract

Although immunotherapy has made great strides in cancer therapy, its effectiveness varies widely among individual patients as well as tumor types, and there is an urgent need to develop biomarkers for effectively assessing immunotherapy response. In recent years, RNA methylation regulators have demonstrated to be novel potential biomarkers for prognosis as well as immunotherapy of cancers, such as N6-methyladenine (m6A) and 5-methylcytosine (m5C). N7-methylguanosine (m7G) is a prevalent RNA modification in eukaryotes, but the relationship between m7G regulators and prognosis as well as tumor immune microenvironment is still unclear. In this study, a pan-cancer analysis of 26 m7G regulators across 17 cancer types was conducted based on the bioinformatics approach. On the one hand, a comprehensive analysis of expression features, genetic variations and epigenetic regulation of m7G regulators was carried out, and we found that the expression tendency of m7G regulators were different among tumors and their aberrant expression in cancers could be affected by single nucleotide variation (SNV), copy number variation (CNV), DNA methylation and microRNA (miRNA) separately or simultaneously. On the other hand, the m7Gscore was modeled based on single sample gene set enrichment analysis (ssGSEA) for evaluating the relationships between m7G regulators and cancer clinical features, hallmark pathways, tumor immune microenvironment, immunotherapy response as well as pharmacotherapy sensitivity, and we illustrated that the m7Gscore exhibited tight correlations with prognosis, several immune features, immunotherapy response and drug sensitivity in most cancers. In conclusion, our pan-cancer analysis revealed that m7G regulators may exert critical roles in the tumor progression and immune microenvironment, and have the potential as biomarkers for predicting prognosis, immunotherapy response as well as candidate drug compounds for cancer patients.

## 1 Introduction

Immunotherapies such as immune checkpoint blockade (ICB) have exerted revolutionary influence on cancer treatment, particularly in the end-stage patients in the last decade (Zhu et al., 2021). Immunotherapies have shown strong antitumor activity in multiple solid tumors, such as non-small cell lung cancer, prostate cancer and melanoma, by means of re-awakening and enhancing the anti-tumor immunity (Guha et al., 2022). Nevertheless, the effectiveness of immunotherapy varies greatly among different populations, tumors, and individuals, and only a fraction of patients benefit from the treatment (Wang et al., 2021). Studies have demonstrated that tumor immune microenvironment (TIME) plays essential roles in the pathogenesis of cancer, and its heterogeneity determines the immunotherapeutic effect to a certain extent (Bai and Cui, 2022). Therefore, significant efforts have been made to identify reliable predictive biomarkers of response and resistance to immunotherapy.

RNA epigenetic modifications, such as N6-methyladenine (m6A) and 5-methylcytosine (m5C), was closely related to tumor genesis and progression, and the corresponding regulators performed well in predicting tumor prognosis and immunotherapy response (Guo et al., 2021; Pan et al., 2021; Zhu et al., 2021; Huang et al., 2022). N7-methylguanosine (m7G) is another pattern of RNA modification, which methylate the N7-atom of guanine (G) by methyltransferase, such as METTL1 (Cheng et al., 2022). The m7G modification often occurred in the 5′cap and internal positions of messenger RNA or internally within ribosomal RNA and transfer RNA (Shoombuatong et al., 2022). Besides, m7G modification has recently been found in primary microRNA (pri-miRNA) and long noncoding RNA (lncRNA) (Pandolfini et al., 2019; Wang et al., 2022). M7G modification mainly exerts their biological functions by regulating RNA processing and metabolism, involving transcription elongation, translation, splicing, polyadenylation, nuclear export, tRNA stability, rRNA maturation and miRNA biosynthesis (Luo et al., 2022). To date, several m7G regulators (mainly m7G methyltransferases) have been revealed to be aberrantly expressed in cancers and regulate tumor-related biological functions through mediating m7G modification of tRNA or miRNA, suggesting m7G modification may exert fundamental effects in tumor genesis and progression like other RNA methylation modifications such as m6A and m5C. For example, METTL1, a m7G methyltransferase, its aberrant upregulation in tumors has been found to be linked to end-stage tumors and worse prognosis, and METTL1 can drive oncogenic transformation and accelerate tumor progression by promoting m7G tRNA modification (Chen et al., 2021; Ma et al., 2021; Orellana et al., 2021). Notably, recent studies have uncovered that risk models based on m7G-associated miRNA and lncRNAs have potential value in predicting tumor prognosis and immunotherapy outcomes (Hong et al., 2022; Zhang et al., 2022). However, the potential of m7G regulators to serve as predictive biomarkers of prognosis and immunotherapy response across cancers remains unclear.

In the present study, we performed a pan-cancer analysis of 26 m7G regulators across 17 cancer types using The Cancer Genome Atlas (TCGA) datasets. Initially, a comprehensive analysis of expression features, genetic variations and epigenetic regulation of m7G regulators was carried out; next, the m7Gscore was modeled based on the single sample gene set enrichment analysis (ssGSEA) and the relations between m7G regulators and cancer clinical features, tumor immune microenvironment, immunotherapy response as well as pharmacotherapy sensitivity were then dissected. In brief, our integrative analysis may provide a new perspective into molecular mechanisms of m7G modification and lay a theoretical support for m7G regulators as biomarkers for prognosis, and response to immunotherapy and chemotherapy in human cancers.

## 2 Material and methods

### 2.1 Source and datasets

The mRNA expression raw counts data and corresponding clinical data were downloaded from TCGA (https://portal.gdc.cancer.gov/) and different normal tissues from healthy subjects were obtained from GTEx dataset (https://commonfund.nih.gov/GTEx), including 7,862 samples and 31 tissues. The TPM normalized gene expression data, copy number variation data estimated using the GISTIC2 threshold method, DNA methylation data (Methylation450K), somatic mutation data and miRNA expression data of pan-cancer tumor and normal patients were achieved from UCSC Xena browser (https://portal.gdc.cancer.gov/). The immunophenoscore was calculated based on The Cancer Immunome Atlas (TCIA, https://www.tcia.at/home) which can also be queried for the cellular composition of immune infiltrates, cancer-germline antigens and the expression of specific immune-related gene sets. The dysfunction and exclusion score of patients was calculated based on Tumor Immune Dysfunction and Exclusion (TIDE, http://tide.dfci.harvard.edu/) to predict anti-PD1 and anti-CTLA4 response. The Genomics of Drug Sensitivity in Cancer (GDSC, https://www.cancerrxgene.org/) database was used to explore the drug sensitivity based on 1,000 human cancer cell lines and related 100s of compounds.

In this study, a total of 17 cancer types were included to conduct pan-cancer analysis, involving breast invasive carcinoma (BRCA, *n* = 1,204), kidney renal clear cell carcinoma (KIRC, *n* = 602), lung adenocarcinoma (LUAD, *n* = 572), thyroid carcinoma (THCA, *n* = 563), head and Neck squamous cell carcinoma (HNSC, *n* = 562), lung squamous cell carcinoma (LUSC, *n* = 548), prostate adenocarcinoma (PRAD, *n* = 547), stomach adenocarcinoma (STAD, *n* = 450), bladder urothelial carcinoma (BLCA, *n* = 426), liver hepatocellular carcinoma (LIHC, *n* = 419), colon adenocarcinoma (COAD, *n* = 327), kidney renal papillary cell carcinoma (KIRP, *n* = 320), esophageal carcinoma (ESCA, *n* = 194), uterine corpus endometrial carcinoma (UCEC, *n* = 193), rectum adenocarcinoma (READ, *n* = 101), kidney chromophobe (KICH, *n* = 91), cholangiocarcinoma (CHOL, *n* = 45).

### 2.2 Gene expression pattern in normal tissues

The baseline expression level of m7G regulators in 31 normal tissues were examined based on the GTEx data. 31 normal tissues included heart, blood, brain, kidney, liver, pancreas, muscle, stomach, colon, pituitary, blood vessel, small intestine, adrenal gland, salivary gland, adipose tissue, lung, prostate, esophagus, breast, ovary, nerve, fallopian tube, bladder, spleen, thyroid, uterus, vagina, cervix uteri, testis, skin, and bone marrow. Raw counts were normalized by the method of transcripts per million (TPM).

### 2.3 Differential analysis of gene expression in cancers

Among all the cancers, the number of tumor subjects ranged from 36 to 1,091, while the number of normal subjects ranged from 0 to 113 and we only included 17 cancers which had over ten subjects both in tumor and normal. Then, we performed differential analysis using R package “DESeq2” and obtained the fold change (FC) and adjusted *p*-value (FDR) of each gene in all the 17 cancers. Differential expressed genes with FDR < 0.05 were screened for the following analysis.

### 2.4 Survival analysis of m7G regulators across cancers

After filtering out uncensored data, we performed survival analysis of m7G regulators for the subjects which had both expression data and clinical data in 17 cancers. Tumor subjects were categorized into the high-expression and low-expression group according to the optimal cut-off value of the gene TPM value which was calculated by “surv_cutpoint” function using R package “survminer”. The Kaplan-Meier survival analysis was conducted based on logrank test using R package “survival” for each m7G regulator. Genes with *p*-value < 0.05 were screened for the following analysis.

### 2.5 Single nucleotide variation and mutation analysis of m7G regulators across cancers

Single nucleotide variation (SNV) and mutation data (*n* = 9,104) in pan-cancer was obtained from UCSC Xena browser. After filtering out non-coding region mutations, such as 3′UTR, 5′ UTR, Silent, 3′Flank, and 5′Flank, the mutation frequency of each m7G regulator in 17 cancers were computed. The oncoplot was drawn for showing the mutation pattern of m7G regulators by using R package “maftools”.

### 2.6 Copy number variation analysis of m7G regulators across cancers

The pan-cancer gene-level copy number variation (CNV) data (*n* = 10,845) estimated by GISTIC2 method was downloaded from UCSC Xena browser. The value of CNVs was divided into amplification and deletion according to the threshold of 0.05, and the percentage of different CNV types was subsequently calculated. Homozygous amplification and deletion data were used to assess the relationship between the CNV and the expression level of m7G regulators in 17 cancers using Spearman’s correlation analysis.

### 2.7 DNA methylation analysis of m7G regulators across cancers

The Methylation 450K data (*n* = 9,639) in pan-cancers was downloaded from TCGA database. The value of DNA methylation was calculated by the median of all beta-values obtained from mapped CpG islands in promoter regions, such as TSS150 and TSS200. Differential analysis was exerted to explore the methylation level of m7G regulators in cancer and normal subjects using R package “edgeR” across 17 cancers. Hypomethylated gene and hypermethylated gene was screened by the threshold of *p*-value < 0.05. What’s more, the Spearman’s correlation between the methylation and expression level of m7G regulators was also evaluated.

### 2.8 MicroRNA regulatory network of m7G regulators across cancers

The potential interactions between miRNA and m7G regulator mRNA was evaluated by star base (https://starbase.sysu.edu.cn/). The pan-cancer miRNA expression data (*n* =10,818) was obtained from UCSC Xena browser. The Spearman’s correlation between the expression of m7G regulators and predicted miRNAs was performed and filtered by the threshold of *p*-value < 0.01 and R < −0.25. Subsequently, the miRNA-mRNA regulatory network of m7G regulators was visualized by Cytoscape software.

### 2.9 Multivariate regression analysis of m7G regulator gene expression

The contributions of CNV alteration, DNA methylation and miRNAs dysregulation to the aberrant expression of m7G regulators were assessed by multivariate regression analysis. The expression of m7G regulators was modeled based on the median miRNA expression, median methylation levels, and CNV values of each m7G regulator.

### 2.10 Establishing and evaluating of the m7Gscore

To establish an index to represent the role of m7G regulators, we conducted single-sample gene-set enrichment analysis (ssGSEA) based on the expression of m7G-related gene set. The enrichment scores (ES) of the m7G-related gene set in each subject across cancers were calculated using R package “GSVA”. The m7Gscore between normal and tumor subjects in 17 cancers was estimated and the differential analysis was performed using *t*-test. The *p*-value was adjusted by FDR.

### 2.11 Clinical relevance of the m7Gscore

We stratified the tumor subjects into the high-risk and low-risk groups according to the median of m7Gscore in each tumor. Kaplan-Meier survival analysis was performed to explore the survival difference in overall survival (OS) and disease-specific survival (DSS) between the high-risk and low-risk groups in each cancer. For cancers with *p*-value < 0.05, the survival independence of the m7Gscore as well as clinicopathologic factors (gender, age, race, grade, T, N, M, and tumor stage) was evaluated by performing univariate and multivariate Cox regression analysis. To further evaluate the prognostic value of m7Gscore in cancers with *p*-value < 0.05, we stratified the clinicopathologic features according to grade, stage, T, N, and M and analyzed the difference of DSS between the two risk groups.

### 2.12 Pathway analysis of the m7Gscore

To clarify the pathways related to m7G regulators, we stratified the tumor subjects in each cancer into the m7G-high group (top 30%) and the m7G-low group (bottom 30%) according to m7Gscore. Then we performed gene set enrichment analysis (GSEA) between the m7G-high and m7G-low groups and analyzed the enrichment of hallmark gene sets.

### 2.13 Immune microenvironment and immunotherapy response analysis of the m7Gscore

To explore the role of m7Gscore in tumor immune microenvironment (TIME), we analyzed Spearman’s correlation between m7Gscore and immune parameters, such as immune cell types, immune checkpoint molecules and immunophenoscores (IPSs). For immune cell types, we conducted ssGSEA to quantify the infiltration degree of 28 immune cell types in each subject across cancers using R package “GSVA”, and analyzed the immune cell composition among cancers based on TCIA database. For the immune checkpoint molecules, including PDCD1, CD274, PDCD1LG2, CTLA4, CD276, TNFRSF9, TNFRSF4, TGFB1, CXCR4, LAG3, ADORA2A, ICOSLG, IL1A, IL6, CCL2, IL10, TNFSF4, HAVCR2, CD4, ICOS, TIGIT, and SIGLEC15, their expression differences between the m7Gscore high-risk and low-risk groups were evaluated. For IPS, we downloaded the IPS data from The Cancer Immunome Atlas (TCIA) and assessed their correlation with m7Gscore among cancers. To further investigate the role of m7Gscore in predicting tumor immunotherapy response, the dysfunction and exclusion score of patients was calculated by Tumor Immune Dysfunction and Exclusion (TIDE) database, and Spearman’s correlation analysis between m7Gscore and T cell dysfunction score and exclusion score was conducted.

### 2.14 The potential pharmacotherapy sensitivity prediction of the m7Gscore

The Genomics of Drug Sensitivity in Cancer (GDSC) database was used to explore the drug sensitivity, and the half-maximal inhibitory concentration (IC50) of each compound for patients was calculated by R package “pRRophetic”. To identify novel candidate drug compounds, we performed correlation analysis of m7Gscore and IC50 of each compound for patients across 17 cancers. The potential pharmacotherapy sensitive drugs were filtered by the threshold of *p*-value < 0.05 and R < −0.2/R > 0.2.

## 3 Results

### 3.1 Aberrant expression and clinical relevance of m7G regulators across cancers

**FIGURE 1:**
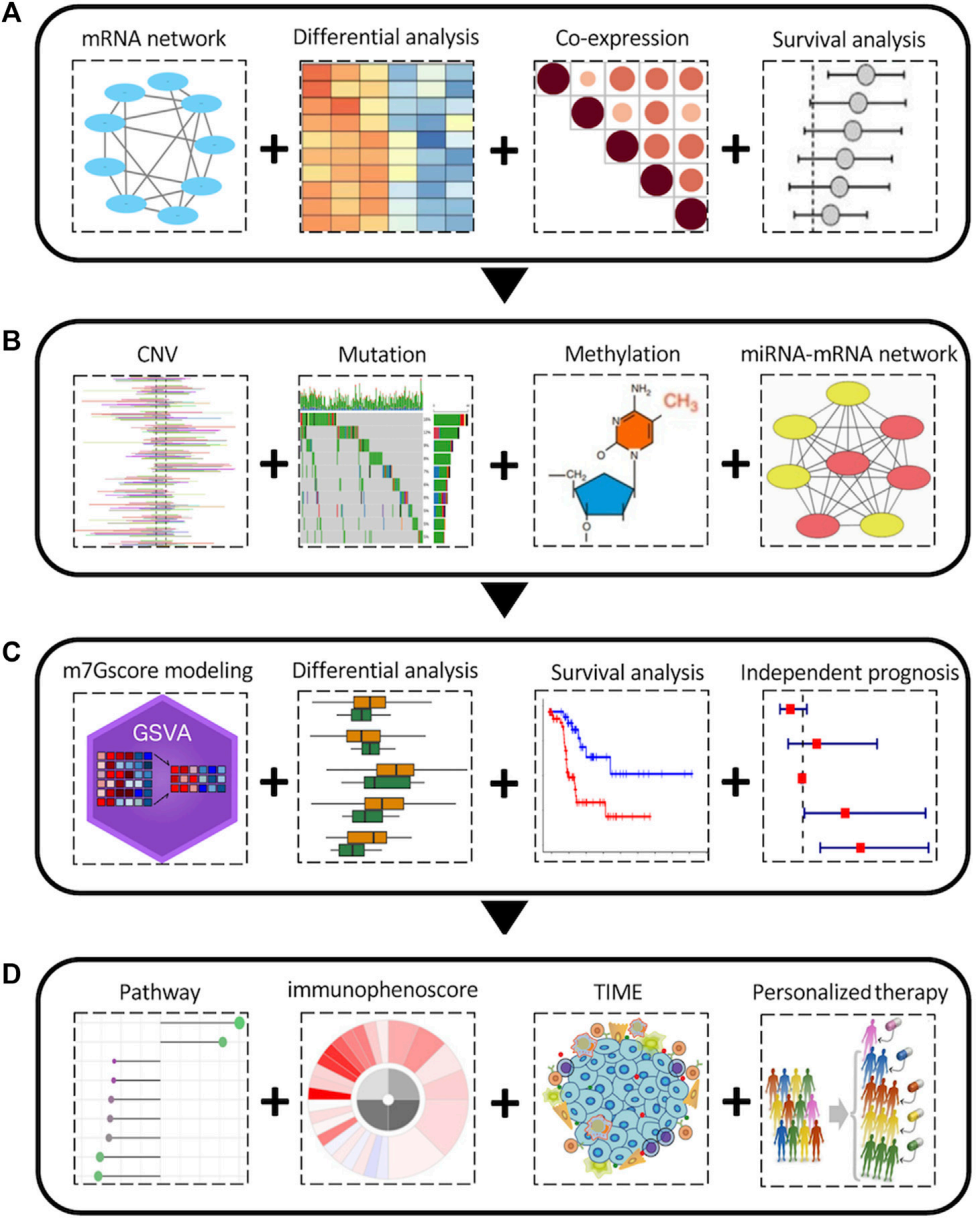
The overall pan-cancer analysis workflow of the m7G regulators. **(A)** Aberrant expression and clinical relevance of m7G regulators across cancers. **(B)** The genetic variations and epigenetic regulation of m7G regulators across cancers. **(C)** m7Gscore modeling and its clinical relevance among cancers. **(D)** Relationship between m7Gscore and associated pathways, immunophenotypes, tumor immune microenvironment, immune therapy and chemotherapy sensitivity among cancers.

**FIGURE 2:**
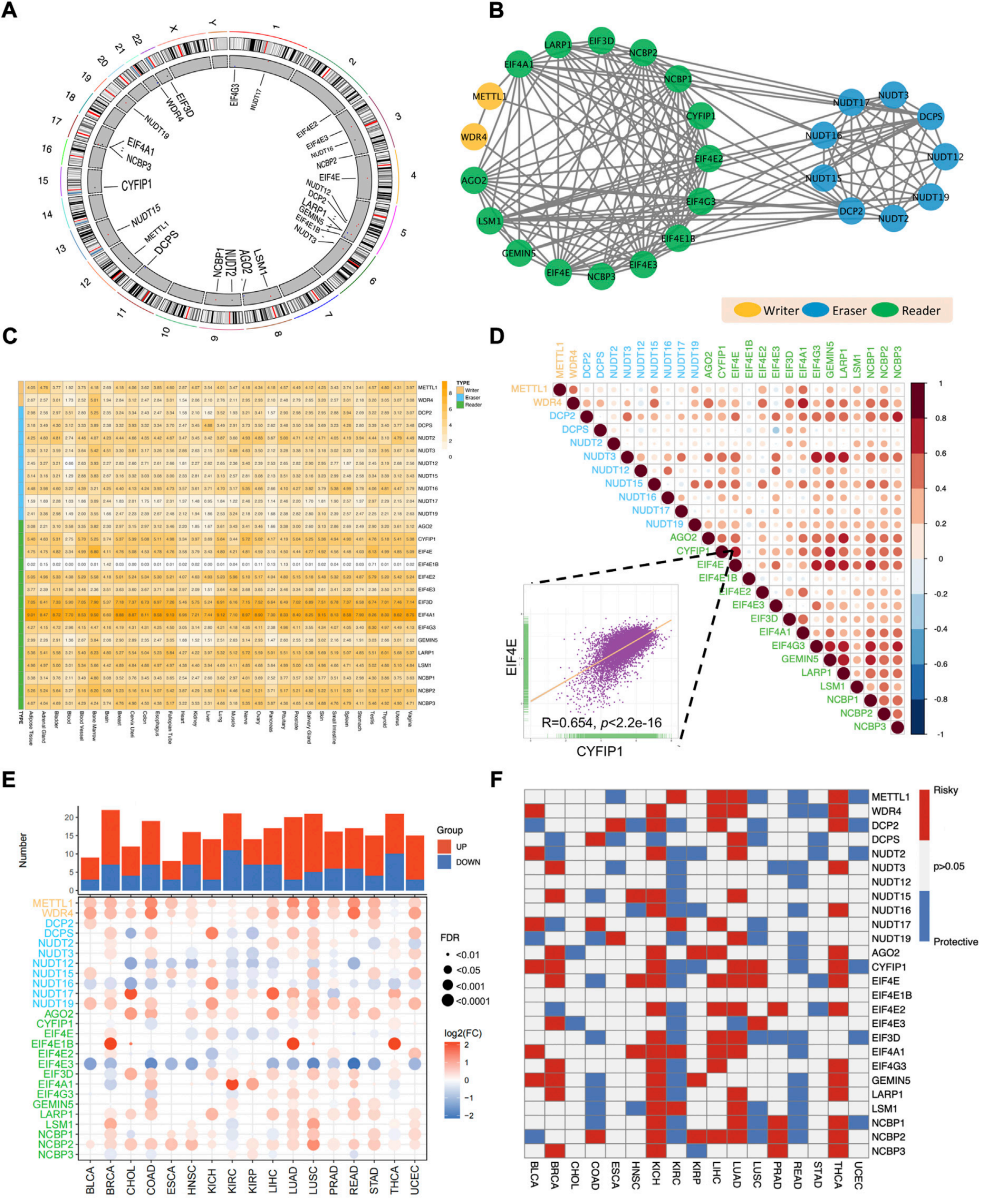
Aberrant expression and clinical relevance of m7G regulators across cancers. **(A)** The circular plot demonstrating the distribution of m7G regulators in human chromosomes. **(B)** The protein-protein interaction network among m7G regulators. **(C)** The expression pattern of m7G regulators among normal tissues in GTEx database. **(D)** The co-occurrence among m7G regulators. The scatter plot indicated the correlation between EIF4E and CYFIP1. **(E)** The differential expression of m7G regulators between tumor and normal tissues across 17 TCGA cancers. **(F)** The clinical relevance of m7G regulators among cancers.

The overall workflow of this pan-cancer analysis of m7G regulators was demonstrated in Figure 1. Initially, we identified 26 m7G regulators and categorized into 3 groups, including 2 writers, 9 erasers, and 15 readers, and their distribution on human chromosomes was displayed by a circular plot (Figure 2A). To explore the collaboration among m7G regulators, we constructed the protein-protein interaction network and found that writer, reader and eraser proteins had high interaction with each other, especially among readers and erasers (Figure 2B). The expression status of m7G regulators among normal tissues was also explored using GTEx data, and the results showed that EIF4A1 and EIF3D had highest expression among different tissues, while EIF4E1B had the lowest expression (Figure 2C). Moreover, a correlation analysis was then performed to investigate the co-occurrence among m7G regulators, and the results showed that most m7G regulators had positively correlated expression patterns, especially among readers and erasers (Figure 2D). For instance, the eraser NUDT3 had high correlation with readers, such as EIF4G3, GEMIN5 and LARP1 and the reader EIF4E had high correlation with readers, such as EIF4G3, GEMIN5, LARP1, and NCBP1.

Next, for the sake of investigating the aberrant expression pattern of m7G regulators across cancers, the expression differences of m7G regulators between tumor and corresponding normal tissues across 17 TCGA cancer types was analyzed. We found that most m7G regulators were significantly upregulated in pan-cancers, especially in LUAD, LUSC, BRCA and UCEC (Figure 2E). What’s more, the expression of some writer genes (METTL1 and WDR4) and reader genes (AGO2 and NCBP2) was significantly upregulated in most cancers, while the expression of some eraser genes (NUDT12 and NUDT16) and some reader genes (EIF4E3) were downregulated in most cancers (Figure 2E).

Obvious aberrant expression of m7G regulators prompted us to investigate their clinical relevance across cancers (Figure 2F). Several cancers showed consistent clinical significance of m7G regulators. Most m7G regulators were survival risky in BRCA, KICH, LIHC, LUAD and THCA, while most regulators were survival protective in KIRC and READ. Furthermore, the m7G regulators had heterogenous cancer type-specific clinical relevance. For example, EIF4A1 was survival risky in several tumors including HNSC, KICH, KIRC, LIHC and LUAD, but showed survival protective in READ. Of note, most m7G regulators functioned survival risky were upregulated in LIHC and most m7G regulators functioned survival protective were downregulated in KIRC. Taken together, those results demonstrated that the collaborative m7G regulators were dysregulated across cancer and may play essential roles in tumorigenesis and progression.

### 3.2 The genetic variations of m7G regulators across cancers

**FIGURE 3:**
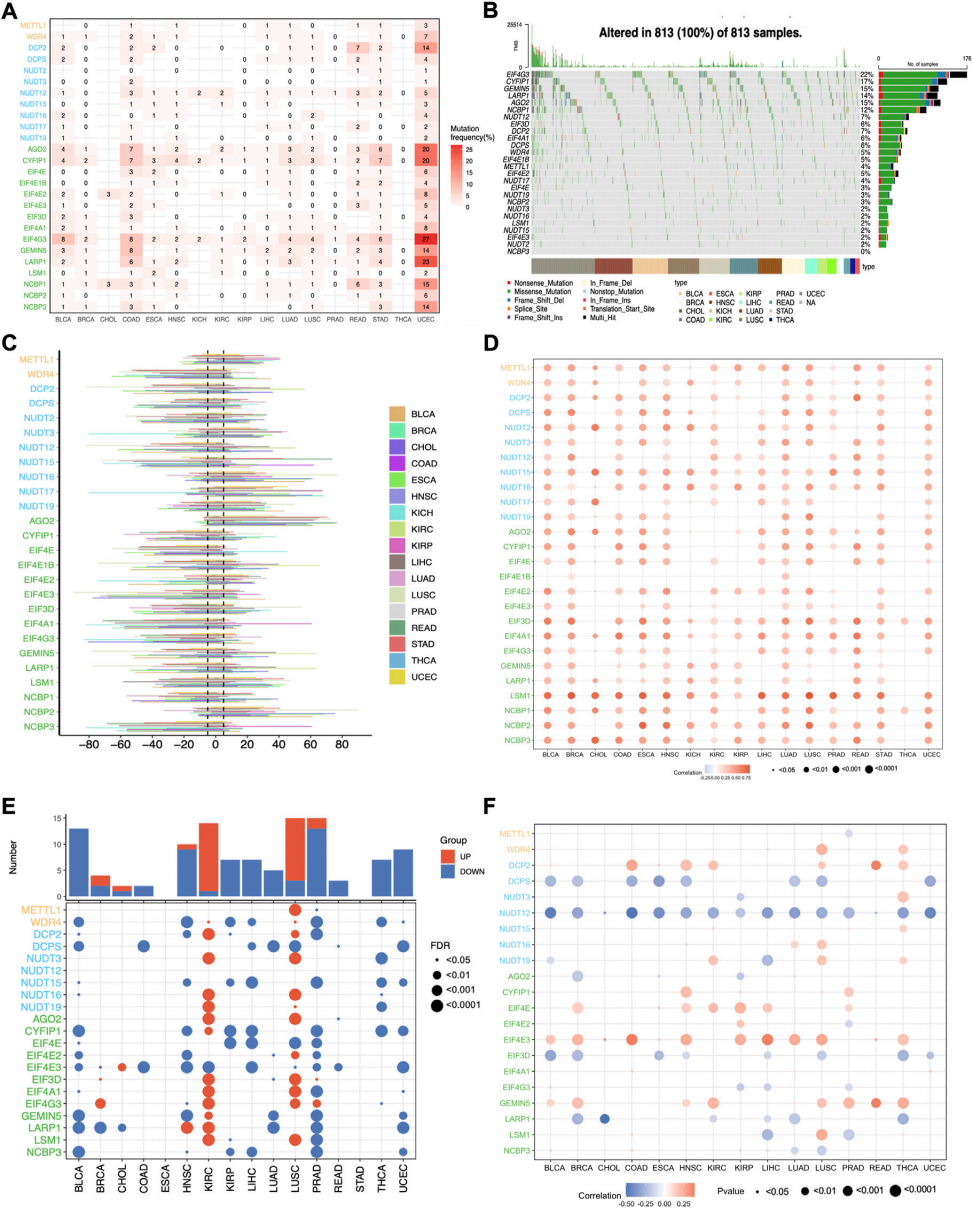
The genetic variations of m7G regulators across cancers. **(A)** The mutation frequency of m7G regulators across cancers. **(B)** The oncoplot shows the mutation pattern of m7G regulators. **(C)** The histogram shows the CNV alteration for each m7G regulators across cancers. **(D)** The Spearman’s correlation between the CNV alterations and the expression of m7G genes. **(E)** Differential analysis of the methylation level of m7G regulators. **(F)** The Spearman’s correlation between the methylation level and expression of m7G genes.

To further dissect the potential molecular mechanism of the aberrant expression of m7G regulators, we examined the SNV data of the 17 selected cancers to calculate the mutation frequency and patterns across cancers, and the results indicated that the mutation frequency varied 0%–10% in most cancers, except for UCEC (Supplementary Figure S1A). The top 5 mutated m7G regulators in UCEC were EIF4G3, LARP1, AGO2, CYFIP1, and NCBP1 with the mutation percentages of 27%, 23%, 20%, 20%, and 15% respectively (Figure 3A). The mutation pattern of all the m7G regulators showed the missense mutations dominated type (Figure 3B). The top 10 mutated regulators across cancers were EIF4G3, CYFIP1, GEMIN5, LARP1, AGO2, NCBP1, NUDT12, EIF3D, DCP2, and EIF4A1 with the mutation frequency of 22%, 17%, 15%, 15%, 14%, 12%, 7%, 7%, 6%, and 6% respectively.

Since CNV alteration can affect gene expression and plays fundamental roles in cancers (Shao et al., 2019), we also analyzed the CNV data of m7G regulators and found that the CNV alteration frequency was higher than 5% in most cancers (Figure 3C). What’s more, different m7G regulators had diverse CNV alteration patterns. METTL1, NUDT16, NUDT17, AGO2, and NCBP2 were characterized as heterozygous amplification, while WDR4, NUTD2, NUTD12, NUTD15, CYF1IP1, EIF4E, EIF4E3, EIF3D, EIF4A1, EIF4G3, and NCBP3 showed heterozygous deletion. The distribution of main CNV alteration patterns across cancers was also revealed by pie plots (Supplementary Figure S1B–D). Correlation analysis demonstrated that the expression of m7G regulators was positively correlated with their CNV alterations, especially LSM1 in most cancers, whereas the correlation of most m7G regulators in THCA showed weakly (Figure 3D). Thus, the above results indicated that the CNV alteration pattern in most cancers may contribute to the aberrant m7G gene expression.

Apart from CNV alteration, DNA methylation, as a critical epigenetic code, also can contribute to tumorigenesis and progression by governing gene expression (Nishiyama and Nakanishi, 2021). Herein, we observed that the methylation pattern of m7G regulators in different cancers is heterogeneous (Figure 3E). Most genes were hypomethylated in BLCA, HNSC, KIRP, LIHC, PRAD, THCA, and UCEC, while most genes were hypermethylated in KIRC and LUSC. What’s more, correlation analysis demonstrated that the expression levels of half of the m7G regulatory factors, such as DCPS, NUDT12, EIF3D, and LARP1, were negatively correlated with methylation levels in most tumors, while the expression of DCP2, EIF4E, EIF4E3, and GEMIN5 showed a positive correlation. (Figure 3F). These results indicate that DNA methylation may contribute to the abnormal expression of some m7G regulators in tumors.

### 3.3 The regulatory network between m7G regulators and microRNAs in cancers

**FIGURE 4:**
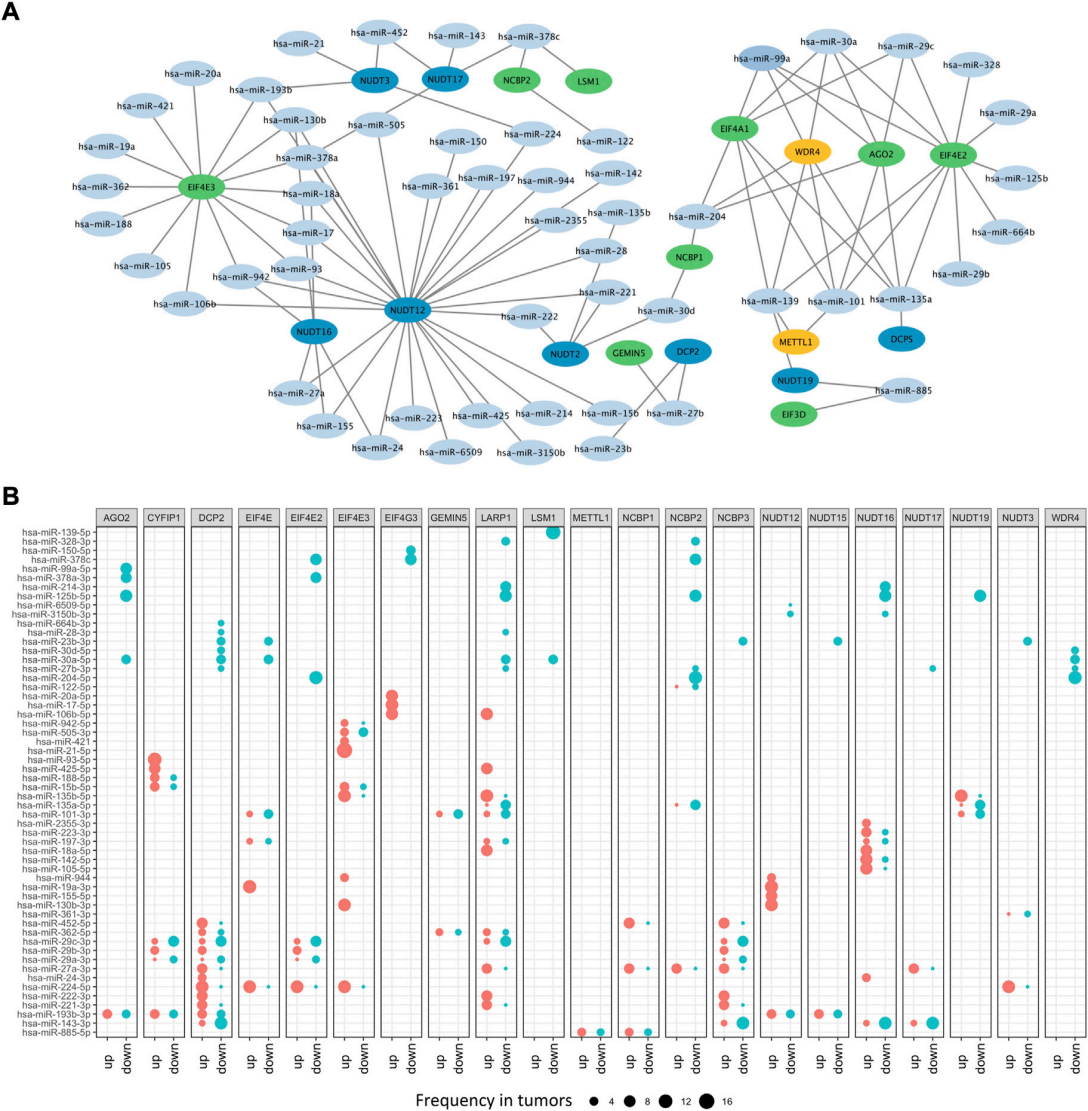
The microRNA network of m7G regulators. **(A)** The miRNA-mRNA network of m7G regulators. **(B)** The differential expressed m7G regulators-related miRNAs.

Besides DNA methylation, miRNA, another important epigenetic regulation mechanism, can modulate gene expression at post-transcriptional level. To illustrate potential m7G regulators-related miRNAs, a m7G regulators-miRNA network was constructed and 56 potential miRNAs targeting 21 m7G regulators screened based on ENCORI database, which can identify miRNA-mRNA interactions under pan-cancer analysis and check whether their expression is negatively correlated (Li et al., 2014). Here, we screened the potential miRNA-m7G regulators interactions existed over six cancer types, and then obtained their regulatory network. The network demonstrated that most m7G regulators could be regulated by miRNAs and some regulators could be targeted by multiple miRNAs, such as NUDT12, EIF4E3, EIF4A1, WDR4, AGO2, and EIF4E2 (Figure 4A). What’s more, differential analysis of potential miRNAs based on miRNA-RNA interactions across cancers were performed, and results indicated that most miRNAs had diverse regulation patterns in various cancers. For example, hsa-miR-224 which targeting DCP2 was up-expressed in 10 cancers, while down-expressed only in 1 cancer. Furthermore, hsa-miR-99a which targeting AGO2 was only down-expressed in 8 cancers, while hsa-miR-93 which targeting CYFIP1 was only upregulated in 12 cancers (Figure 4B).

To further identify different contributions of CNV alteration, DNA methylation and miRNAs dysregulation to the aberrant expression of m7G regulators, we applied multivariate regression analysis and the results indicated that the expression of m7G regulators was affected in different manners (Supplementary Figure S1E). In general, CNV alteration could positively regulate gene expression of m7G regulators, while DNA methylation and miRNAs dysregulation displayed negative effects. Moreover, the expression of m7G regulators could be regulated by one or multiple genetic variations. For example, WDR4 gene expression was affected only by CNV alteration in 8 tumors, including BLCA, BRCA, CHOL, COAD, KICH, KIRC, READ, and UCEC. MiRNAs dysregulation only regulated AOG2 in KIRC, and THCA. However, all three genetic variations significantly contributed to the expression of NUTD12 in 8 tumors, including BLCA, BRCA, ESCA, KIRC, KIRP, LUAD, PRAD, and UCEC.

### 3.4 Differential analysis and clinical relevance of the m7Gscore across cancers

**FIGURE 5:**
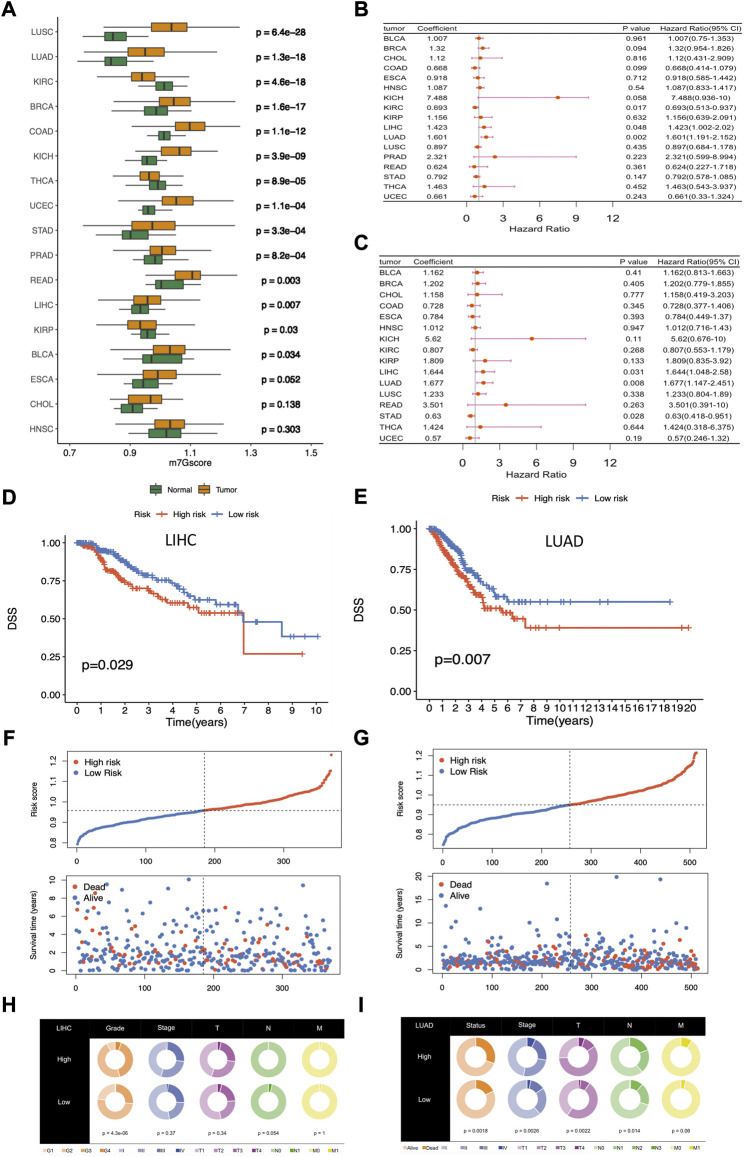
Differential analysis and clinical relevance of the m7Gscore across cancers. **(A)** Differential analysis of m7Gscore between tumor and normal patients across cancers. **(B)** Univariate Cox regression analysis of m7Gscore related to OS across cancers. **(C)** Univariate Cox regression analysis of m7Gscore related to DSS across cancers. **(D)** Kaplan-Meier survival analysis of OS in the two risk groups stratified by m7Gscore in LIHC. **(E)** Kaplan-Meier survival analysis of DSS in the two risk groups stratified by m7Gscore in LUAD. **(F)** Distribution of m7Gscore and survival status in LIHC. **(G)** Distribution of m7Gscore and survival status in LUAD. **(H)** Pie chart of the main clinicopathologic features in the two risk groups in LIHC. **(I)** Pie chart of the main clinicopathologic features in the two risk groups in LUAD.

To further explore the importance of m7G regulators in pan-cancers, we modeled the m7G score by ssGSEA through calculating the normalized enrichment score (NES) of m7G regulator gene sets. Then, differential analysis of m7Gscore between tumor and normal patients across cancers was performed, and results showed that m7Gscore had remarkable differences in most cancers, except for ESCA, CHOL, and HNSC (Figure 5A). Of note, the m7Gscore was only significantly down-expressed in cancers of KIRC, THCA, and KIRP, while up-expressed in the rest cancers.

In the following, we allocated tumor patients into high-risk and low-risk groups based on the median m7Gscore in each cancer and then analyzed the role of m7Gscore in cancer survival. Univariate Cox regression analysis showed that m7Gscore was related to OS in LIHC (HR = 1.423, 95% CI = 1.002–2.020, *p* = 0.048), LUAD (HR = 1.601, 95% CI = 1.191–2.152, *p* = 0.002) and KIRC (HR = 0.693, 95% CI = 0.513–0.937, *p* = 0.017) (Figure 5B), and m7Gscore was also related to DSS in LIHC (HR = 1.644, 95% CI = 1.048–2.580, *p* = 0.031), LUAD (HR = 1.677, 95% CI = 1.147–2.451, *p* = 0.008) and STAD (HR = 0.630, 95% CI = 0.418–0.951, *p* = 0.028) (Figure 5C). The survival analysis indicated that high-risk patients had poor survival in both LIHC (DSS: *p* = 0.029, Figure 5D) and LUAD (DSS: *p* = 0.007, Figure 5E) than low-risk patients. As the figure shown, the mortality was elevated with the increasing of m7Gscore in LIHC and LUAD (Figures 5F,G). What’s more, higher m7Gscore was associated with worser histological grade (*p* < 0.001) in LIHC patients (Figure 5H), while higher m7Gscore was related to worser T (*p* = 0.002), N (*p* = 0.014) and stage (*p* = 0.003) in LUAD patients (Figure 5I). Overall, those results indicated that m7Gscore was closely associated with the malignancy in most cancers and could be a potential novel indicator in predicting prognosis in LUAD and LIHC.

### 3.5 The m7Gscore is an independent prognostic factor in LIHC and LUAD

**FIGURE 6:**
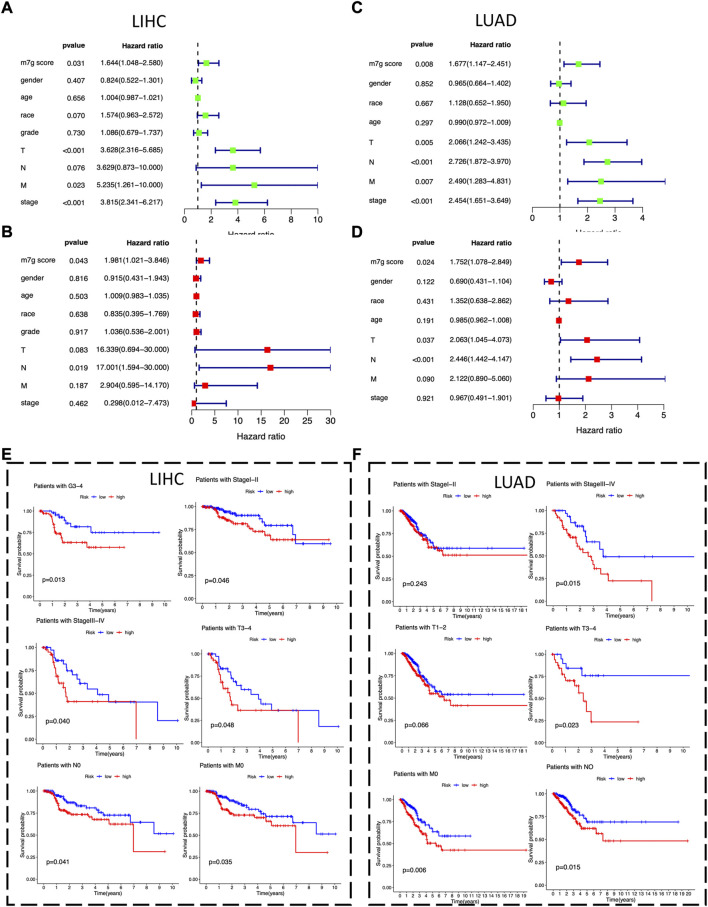
The m7Gscore is an independent prognostic factor in LIHC and LUAD. **(A)** Univariate Cox regression analysis of m7Gscore and main clinicopathologic features associated with DSS in LIHC. **(B)** Multivariate analysis of m7Gscore and main clinicopathologic features associated with DSS in LIHC. **(C)** Univariate Cox regression analysis of m7Gscore and main clinicopathologic features associated with DSS in LUAD. **(D)** Multivariate analysis of m7Gscore and main clinicopathologic features associated with DSS in LUAD. **(E)** Kaplan-Meier survival analysis of DSS stratified by main clinicopathologic features in LIHC. **(F)** Kaplan-Meier survival analysis of DSS stratified by main clinicopathologic features in LUAD.

To identify potential independent prognostic factors in LIHC and LUAD, we exerted univariate and multivariate Cox regression analysis including factors like m7Gscore and main clinicopathologic features, such as gender, age, race, grade, T, N, M, and stage. The results showed that m7Gscore (HR: 1.981, 95% CI = 1.021–3.846, *p* = 0.043) was significantly related to DSS and could be a potential independent prognostic factor in LIHC (Figures 6A,B). Moreover, for LUAD patients, both m7Gscore (HR: 1.752, 95% CI = 1.078–2.849, *p* = 0.024), T (HR: 2.063, 95% CI = 1.045–3.846, *p* = 0.043) and N were potential independent negative prognostic factors of DSS (Figure 6C,D). We subsequently stratified main clinicopathologic features and investigated the prognostic difference of DSS in LIHC and LUAD patients, and the results indicated that the m7Gscore performed well in subgroup of grade 3–4, stage Ⅰ-Ⅱ, stage Ⅲ-Ⅳ, T3-4, N0 and M0 in LIHC patients, while m7Gscore also performed well in subgroup of stage Ⅲ-Ⅳ, T3-4, N0 and M0 in LUAD patients (Figures 6E,F). Taken together, the high-risk patients had poorer prognostic outcomes than low-risk patients.

### 3.6 Relationships between m7Gscore and hallmark pathways among cancers

**FIGURE 7:**
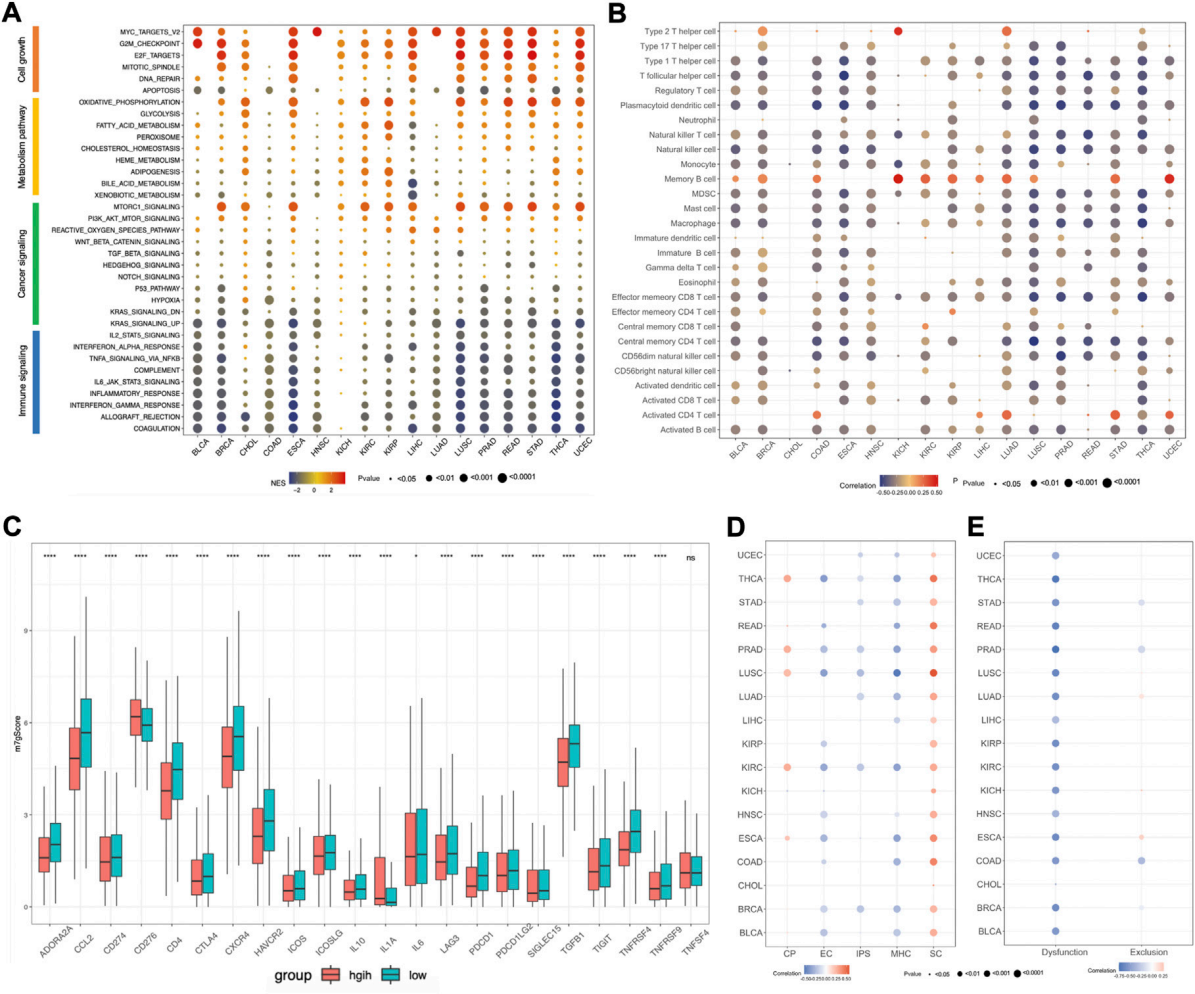
Association between m7Gscore and hallmark pathways, tumor immune microenvironment, immunotherapy response among cancers. **(A)** GSEA for hallmark pathways between top 30% and bottom 30% of m7Gscore in each cancer. **(B)** The correlation between m7Gscore and immune cells among cancers based on ssGSEA. **(C)** The differential analysis of immune checkpoint molecules between m7Gscore high-risk and low-risk groups in pan-cancers. **(D)** The correlation between m7Gscore and immunophenotypes calculated by TCIA database across cancers. **(E)** The correlation between m7Gscore and T cell dysfunction/exclusion score by TIDE database across cancers.

To further explore the relationship between m7Gscore and hallmark pathways among cancers, we performed gene set enrichment analysis (GSEA) based on the two tumor groups, featured as top 30% and bottom 30% of the m7Gscore in each cancer. The hallmark pathways could be categorized into four types, involving cell growth, metabolism, cancer signaling and immune signaling, and we observed that different types of pathways had distinct expression patterns (Figure 7A). For cell growth, pathways related to cell proliferation were enriched in the high-m7Gscore group, while pathways related to cell death were enriched in the low-m7Gscore group. For example, the G2M checkpoint pathway was positively correlated with m7Gscore in 14 cancers, while the apoptosis pathway was negatively correlated with m7Gscore in 15 cancers. For metabolism, oxidative phosphorylation, glycolysis and fatty acid metabolism pathway were activated in the high-m7Gscore group in most cancers. For cancer signaling, MTORC1 and PI3K-AKT-MTOR pathway were significantly enriched in the high-m7Gscore group, while KRAS and hypoxia pathways were enriched in the low-m7Gscore group. For immune signaling, we observed that most immune pathways were inactivated in high-m7Gscore group. For example, IL2-STAT5 signaling, IL6-STAT3 signaling and TNFα signaling were negatively correlated with m7Gscore among cancers. Furthermore, the correlation between the expression of m7G regulators with the NES score of each hallmark pathway in pan-cancers were also analyzed. As shown in Supplementary Figure S2A, METTL1, WDR4, NUDT15, AGO2, EIF4E2, EIF4A1, and NCBP2 had the same expression pattern with m7Gscore in cell growth signaling pathways, while NUDT2, NUDT3, NUDT12, NUDT16, NUDT17, EIF4E1B, LSM1, and NCBP2 had the same expression pattern with m7Gscore in immune signaling pathways.

### 3.7 Association between m7Gscore and tumor immune microenvironment, immunotherapy response among cancers

As there was a negative correlation of m7Gscore and immune signaling pathways, we further explored the roles of m7Gscore in tumor immune microenvironment (TIME) among cancers. For the immune cell types among cancers, we revealed that m7Gscore had inverse correlation with most immune cells, except for activated CD4 T cell, memory B cell and Th2 cell (Figure 7B). And differential analysis indicated that the m7Gscore of activated CD4 T cell, memory B cell and Th2 cell were higher in the high-risk patients (Supplementary Figure S3A). For example, m7Gscore was positively correlated with memory B cell and activated CD4 cell in LIHC, while m7Gscore also positively correlated with memory B cell, activated CD4 T cell and Th2 cell in LUAD (Supplementary Figure S2B,C). Moreover, we also investigated the relationship between the expression of m7G regulators with each immune cell in LIHC and LUAD, and the results showed that Th2 cell, memory B cell and activated CD4 T cell were positively associated with most m7G regulators and EIF4E3 was positively related with most immune cells in both LIHC and LUAD (Supplementary Figure S2D,E). To further validate those results, we also analyzed the immune cell composition among cancers using TCIA database and we also observed that only CD4 T cell was positively correlated with m7Gscore in most cancers (Supplementary Figure S3B). For the immune checkpoint molecules, we performed differential analysis and found that most immune checkpoint molecules were significantly different expressed between the m7Gscore high-risk and low-risk groups, except for TNFSF4 (Figure 7C). Among those different expressed molecules, most molecules were down-regulated in the high-risk group, while only CD276 and IL-1A were up-regulated. What’s more, correlation analysis between the m7Gscore and immune checkpoint molecules in each cancer was also conducted and we found most immune checkpoint molecules were negatively correlated with the m7gscore across cancers, except for CD274 in KIRP, KIRC and KICH, CD276 in PRAD and ESCA, ICOSLG in KIRP and KIRC, IL-1A in HNSC, SIGLEC15 in KICH and TNFSF4 in PRAD (Supplementary Figure S3C).

Previous research found that immunophenoscore (IPS) can be used to evaluate tumor immunogenicity and predict the response to immune checkpoint inhibitor, which was classified into four categories, including MHC molecules (MHC), immunomodulators (CP), effector cells (EC) and suppressor cells (SC) (Charoentong et al., 2017). Herein, our results indicated that the m7Gscore was negative correlated with IPS in half of cancer types, like UCEC, THCA, STAD, PRAD, LUSC, LUAD, KIRC, and BRCA. Moreover, the m7Gscore was positively correlated with SC, but negatively correlated with MHC and EC in most cancers (Figure 7D). Tumor Immune Dysfunction and Exclusion (TIDE) database can be used to predict tumor immunotherapy response based on gene expression matrix (Fu et al., 2020). To further investigate the role of the m7Gscore in predicting tumor immunotherapy response, we conducted correlation analysis and found that the m7Gscore was negatively correlated with the T cell dysfunction score in most cancers, while there was no consistent correlation between the m7Gscore and T cell exclusion score (Figure 7E). What’s more, patients in the immunotherapy responder group had lower m7Gscore in most cancers than the non-responder group, except for LIHC (Supplementary Figure S3D). Patients in the m7Gscore high-risk group had lower immunotherapy responder rate than the low-risk group, except for CHOL and LIHC (Supplementary Figure S3E).

### 3.8 The pharmacotherapy sensitivity prediction based on the m7Gscore

To evaluate the association between m7G regulators and drug sensitivity and identify novel candidate drug compounds, the correlation between the m7Gscore and half-maximal inhibitory concentration (IC50) of each compound for patients across cancers was examined based on GDSC database and the results demonstrated that the IC50 of 62 drugs was remarkable associated with the m7Gscore, involving 43 drugs with positively correlation and 19 drugs with negatively correlation (Supplementary Figure S4). What’s more, the contribution of each m7G regulator to drug sensitivity was investigated and we found that the m7G regulators can be categorized into two groups according to different correlation patterns. On the one hand, most m7G regulators had uniform correlation with IC50 of drugs, such as, the IC50 of PD-0332991 (alias Palbociclib, CDK4/6 inhibitor) and AZD0530 (alias Saracatinib, Src Inhibitor) were uniformly positive correlated with most m7G regulators. On the other hand, some m7G regulators had heterogenous correlation with IC50 of drugs. For example, NUDT12 and NUDT16, which were positively correlated with A.443,654 (Akt inhibitor) and BI-2536 (PLK inhibitor), while other m7G regulators including NUTD19 and NCBP2 had negative correlation. In conclusion, the m7Gscore might be a potential biomarker which could predict candidate drug compounds across cancers.

## 4 Discussion

Growing evidence indicating that RNA epigenetic modification plays fundamental roles in tumorigenesis and progression, and its regulatory genes exhibit great potential as predictor for prognosis and immunotherapy response. For example, m6A, a well-studied RNA modification type, its regulators have been uncovered to be tightly related to prognosis, tumor immune microenvironment, tumor cell stemness, and anticancer drug sensitivity through pan-cancer analysis (Li et al., 2021). M5C, another common RNA modification in eukaryotes, its regulators have also been demonstrated by a pan-cancer analysis to be closely correlated with cancer progression and patient survival (He et al., 2021), and can affect the tumor immune microenvironment in several tumor types (Huang et al., 2021; Pan et al., 2021; Fang et al., 2022). Besides, N1-methyladenosine (m1A), as a critical posttranscriptional RNA modification, its regulators have also recently been revealed to possess potential value as biomarkers for predicting prognosis and evaluating the tumor immune microenvironment (Gao et al., 2021; Zheng et al., 2021; Zhao et al., 2022).

N7-methylguanosine (m7G) is another pattern of RNA modification in post-transcriptional regulation, and it generally occurs in the 5′ cap or internal regions of multiple kinds of RNA, including tRNA, rRNA, mRNA, lncRNA as well as pre-miRNA (Zhang et al., 2019). Like m6A, m5C, and m1A, m7G modification has also been found to be involved in tumor progression (Luo et al., 2022). Interestingly, m7G related lncRNAs have recently been disclosed to be aberrant expressed in several tumors and tightly related to the prognosis and tumor immune microenvironment of patient (Chen S. et al., 2022; Dong et al., 2022; Wu et al., 2022), suggesting that m7G regulators have the potential to predict tumor prognosis and immunotherapy effects. For example, we recently constructed a m7G-related lncRNAs risk model to predict prognosis, immunotherapy response, and drug sensitivity in LIHC (Wei et al., 2022). However, the relationship between m7G regulatory genes and tumor prognosis as well as the immune microenvironment is not yet clear and needs to be further explored. Thus, in this study, a pan-cancer analysis of 26 m7G regulators across 17 cancer types was carried out to survey their expression characteristics and clinical significance in tumors through bioinformatics approach.

First, in our study, we found that the expression trends of m7G regulators and their correlations with survival of patient were different in different tumors, suggesting that m7G modification plays different roles in different tumors. Most obviously, m7G modification might play opposing roles in liver and kidney cancer, as almost all m7G regulators were up-regulated in liver cancer (CHOL and LIHC) and were risk factors for survival, whereas in kidney cancer (KICH, KIRC), nearly all m7G regulators exhibited the low expression and were protective factors for survival, suggesting that m7G modification plays an oncogenic role in liver cancer and play a tumor suppressive role in kidney cancer. Up to now, several m7G regulators have been confirmed to promote liver cancer progression by regulating m7G modification of tRNA, such as METTL1and WDR4 (Chen et al., 2021). Unfortunately, the biological function of the m7G regulators in kidney cancer has not been reported by experimental validation (Dong et al., 2022).

Second, the genetic variations (SNVs and CNVs) and epigenetic regulation (DNA methylation and miRNAs) of m7G regulators were examined to understand the mechanism of their abnormal expression in cancer. For SNV, we found the genetic mutation patterns of m7G regulators were dominated by missense mutations, and any missense mutations in the m7G regulators which are associated with tumor progression have not yet been identified, unfortunately. For CNV, we revealed that there was a close correlation between CNV and differential gene expression of m7G regulators in almost all tumors, suggesting that CNVs could contribute to the abnormal expression of m7G regulators in tumors. For DNA methylation, we disclosed that the DNA methylation patterns of m7G regulators were heterogeneous in different cancers and largely corresponded to their gene expression trends in several cancers. For example, in LIHC, all detectable genes were hypomethylated, while they showed a highly expression status. In KIRC, almost all detectable genes were hypermethylated, whereas they showed a low expression status. For miRNAs, we constructed the network of miRNA-m7G modulators, and found that most m7G regulators could be regulated by miRNAs and some regulators could be targeted by multiple miRNAs, indicating that miRNA as critical epigenetic regulators could participate in the aberrant expression of m7G regulators in tumors. Recent study founded that miR-4293 can promote the proliferation of lung carcinoma by targeting DCP2, which is a mRNA-decapping enzyme (Zhang et al., 2021).

Third, to investigate the roles of m7G regulators, the m7Gscore was established by performing ssGSEA. We found that m7Gscore was significantly down-regulated only in KIRC, THCA and KIRP, while up-regulated in most cancer types including LUSC, LUAD, LIHC etc., indicating that the m7G score was overall consistent with the gene expression trend of m7G regulators in cancers. By conducting survival analysis, the m7Gscore was revealed to be an independent prognostic factor in LUAD and LIHC. A recent study unveiled that a prognostic model containing 7 m7G regulators performed well in predicting survival outcomes in LIHC(Li et al., 2022).

Fourth, the relationship between the m7Gscore and hallmark pathways among cancers was assessed, and we found that the pathways significantly related to the m7Gscore mainly involve cell growth, metabolism, cancer signaling and immune signaling. For cell growth pathway, the results suggested m7G regulators may mainly exert roles in promoting cell proliferation by modulating cell cycle and apoptosis. METTL1, a m7G “writer”, has been proved to promote the proliferation of LIHC cells by accelerating cell cycle G2/M transition and suppressing apoptosis (Chen et al., 2021). For metabolism, the results hint that m7G regulators may positively modulate oxidative phosphorylation, glycolysis and fatty acid metabolism pathways. For cancer pathway, PT3K/AKT/mTORC1 signaling was found to be the most significantly associated with the m7Gscore. Recently, METTL1 was validated to promote the proliferation and autophagy of HNSC cells by up-regulating the PT3K/AKT/mTOR signaling pathway (Chen J. et al., 2022). For immune signaling, we revealed that almost all immune-related pathways were negatively correlated with the m7Gscore, suggesting that m7G regulators may associated with tumor immunosuppressive microenvironment.

Fifth, to further explore the roles of m7G regulators in the TIME, we detailed evaluated the immune parameters, including immune cell types, immune checkpoint molecules, immunophenoscores (IPSs). Herein, results indicated that the m7Gscore was negatively correlated with most immune cells as well as checkpoint molecules in cancers. A recent study showed that the proportion of infiltrating Mrc1+ macrophages, Macro-3 cells and Langerhans cells in HNSC tissues was significantly increased after METTL1 knockdown, while CD4^+^ T exhaustion and regulatory T cells were remarkably decreased (Chen J. et al., 2022). In addition, the m7Gscore was also unveiled to be correlated with poor immunotherapy response in most cancers. Thus, m7G regulators may be the potential biomarkers for predicting tumor immune microenvironment and immunotherapy response.

Finally, the association between the m7G regulators and drug sensitivity was explored based on the GDSC database and totally 62 drugs were disclosed to be significantly associated with the m7G scores, indicating that m7G regulators have the potential as biomarkers for predicting candidate drug compounds for cancer patients.

## 5 Conclusion

Our pan-cancer analysis demonstrated that m7G regulators may play a significant role in the tumor progression and immune microenvironment, and show the potential as biomarkers for predicting prognosis, immunotherapy response as well as candidate drug compounds for cancer patients. Meanwhile, this study will provide novel clues for further basic and clinical translational research of m7G regulators in cancers.

## Data Availability

The original contributions presented in the study are included in the article/Supplementary Material, further inquiries can be directed to the corresponding authors.
