# Supplementary material for: Comprehensive pan-cancer analysis of N7-methylguanosine regulators: Expression features and potential implications in prognosis and immunotherapy
Source: Front Genet. 2022 Oct 21;13:1016797. doi: 10.3389/fgene.2022.1016797 (PMC9633684; doi:10.3389/fgene.2022.1016797)
Supplement: Supplementary file 1 [file DataSheet1.docx]

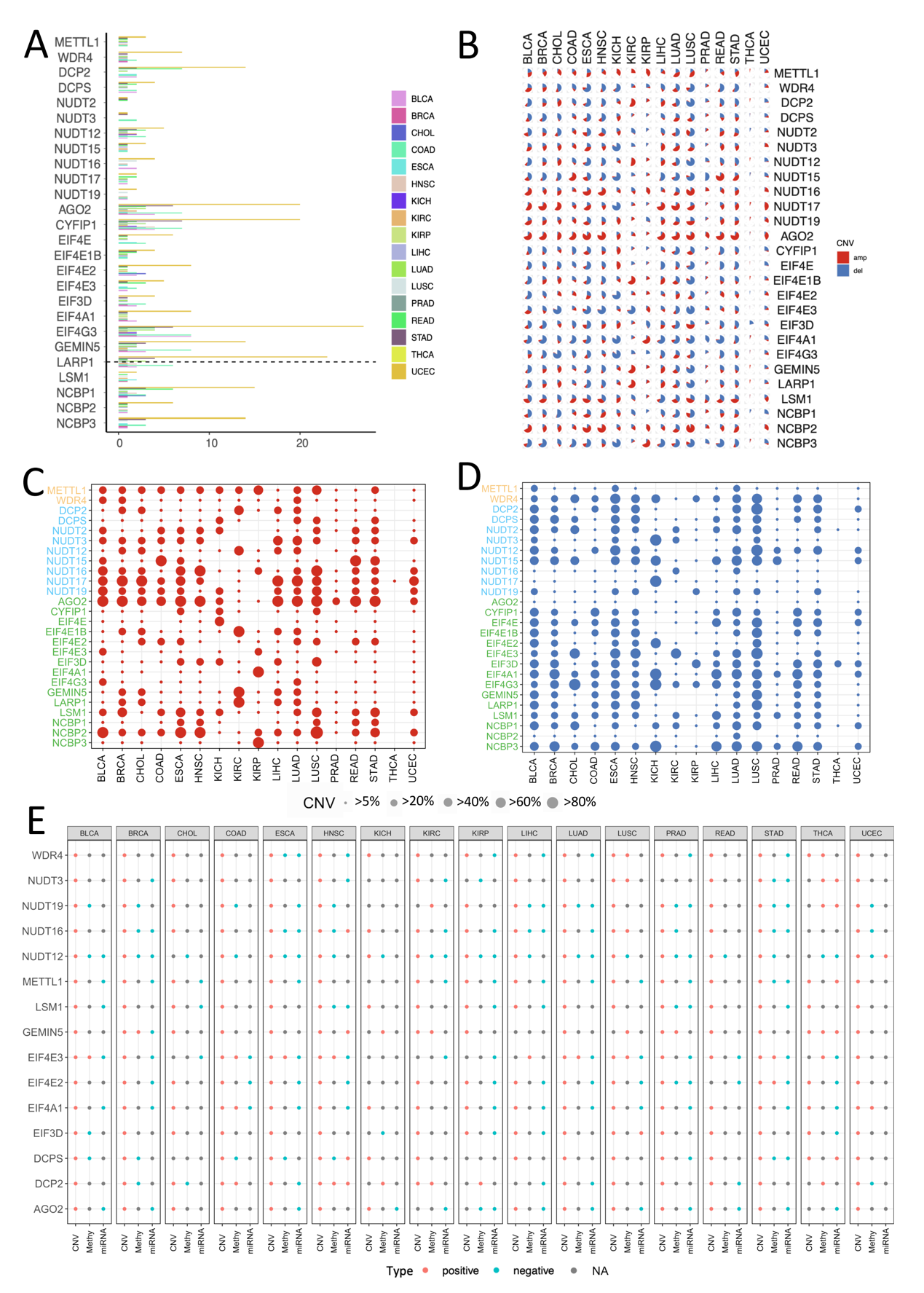


Figure S1. Molecular variations of m7G regulators related to Figure 3 and Figure 4. (A) The histogram shows the mutation frequency of m7G regulators across cancers. (B) The distribution of main CNV alteration patterns of m7G regulators across cancers. (C) The percentage of CNV amplification for each m7G gene across cancers. (D) The percentage of CNV deletion for each m7G gene across cancers. (E) The relationship between the expression of m7G regulators with their CNV alteration, DNA methylation and miRNA expression.


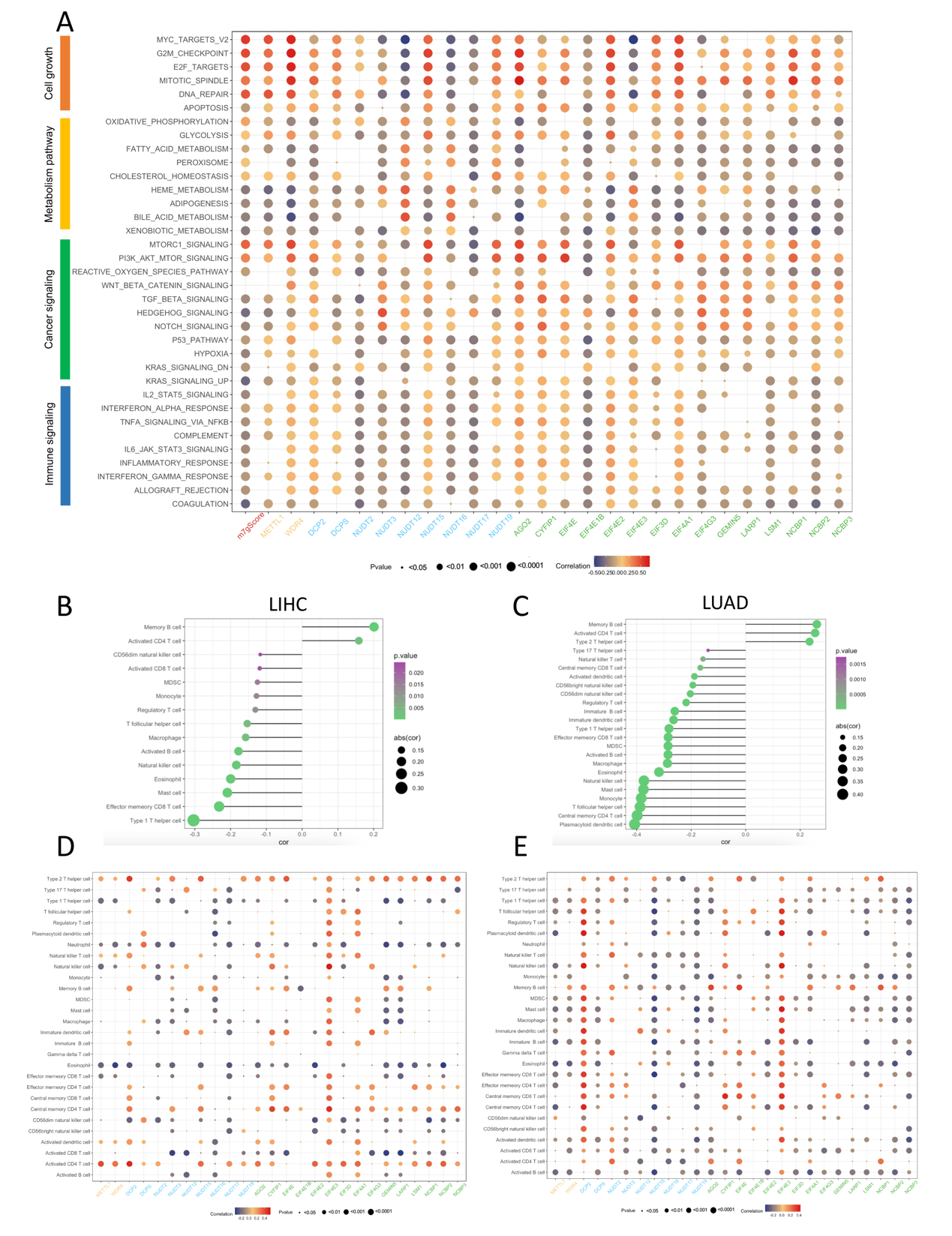


Figure S2. Association between m7Gscore and hallmark pathways, tumor immune cells among cancers related Figure 7. (A) The correlation between the expression of m7G regulators and NES score of each hallmark pathway in pan-cancers. (B-C) The correlation between m7Gscore and immune cells in LIHC and LUAD. (D-E) The correlation between the expression of m7G regulators with NES score of each hallmark pathway in LIHC and LUAD.


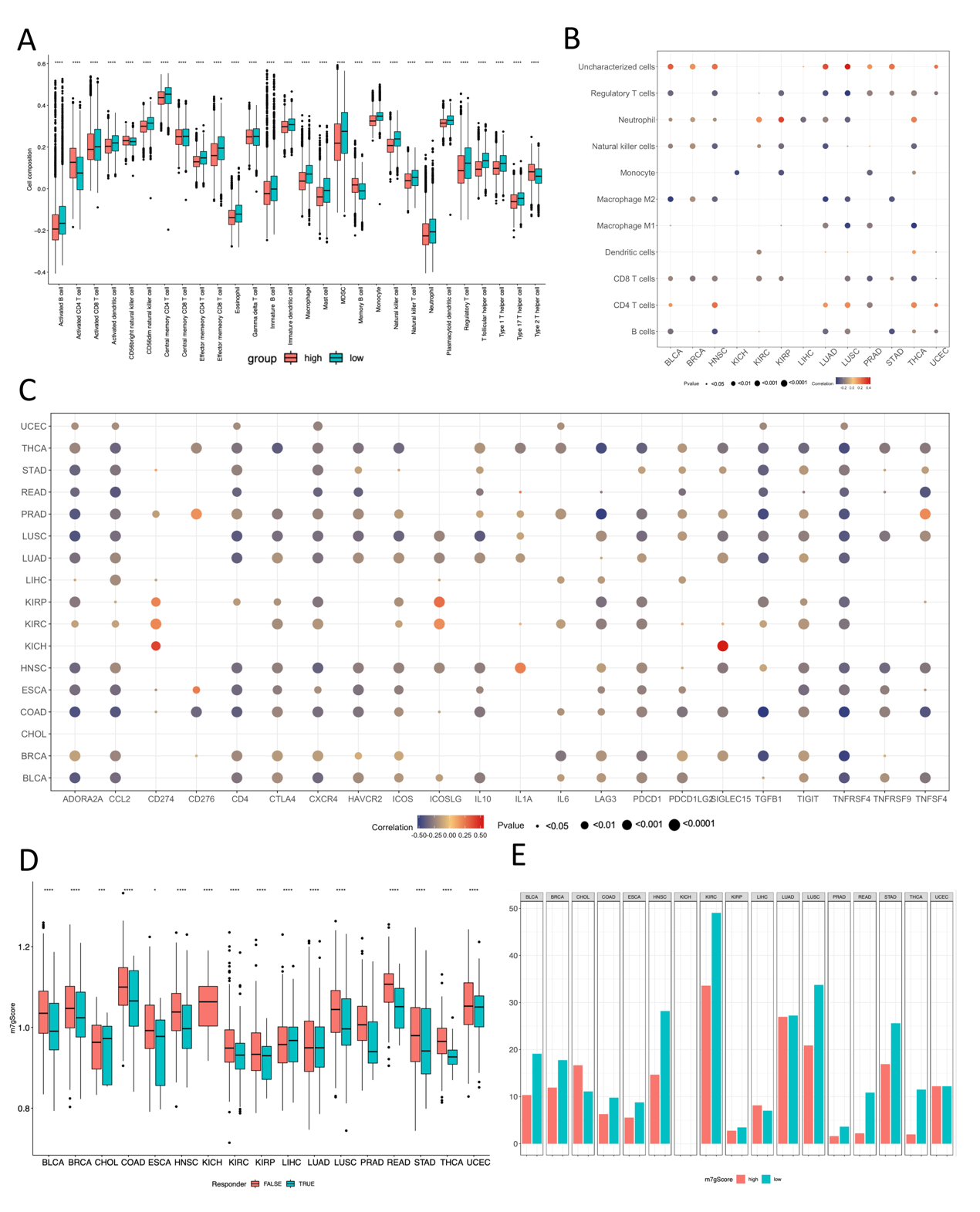


Figure S3. Association between m7Gscore and tumor immune microenvironment, immunotherapy response among cancers related Figure 7. (A) The differential analysis of immune checkpoint molecules expression between m7Gscore high-risk and low-risk groups. (B) The correlation between m7Gscore and immune cells among cancers based on TCIA database. (C) The correlation between m7Gscore and the expression of immune checkpoint molecules in pan-cancers. (D) The differential analysis of m7Gscore between immunotherapy responder and non-responder groups based on TIDE database. (E) The differential responder rate between m7Gscore high-risk and low-risk group based on TIDE database.


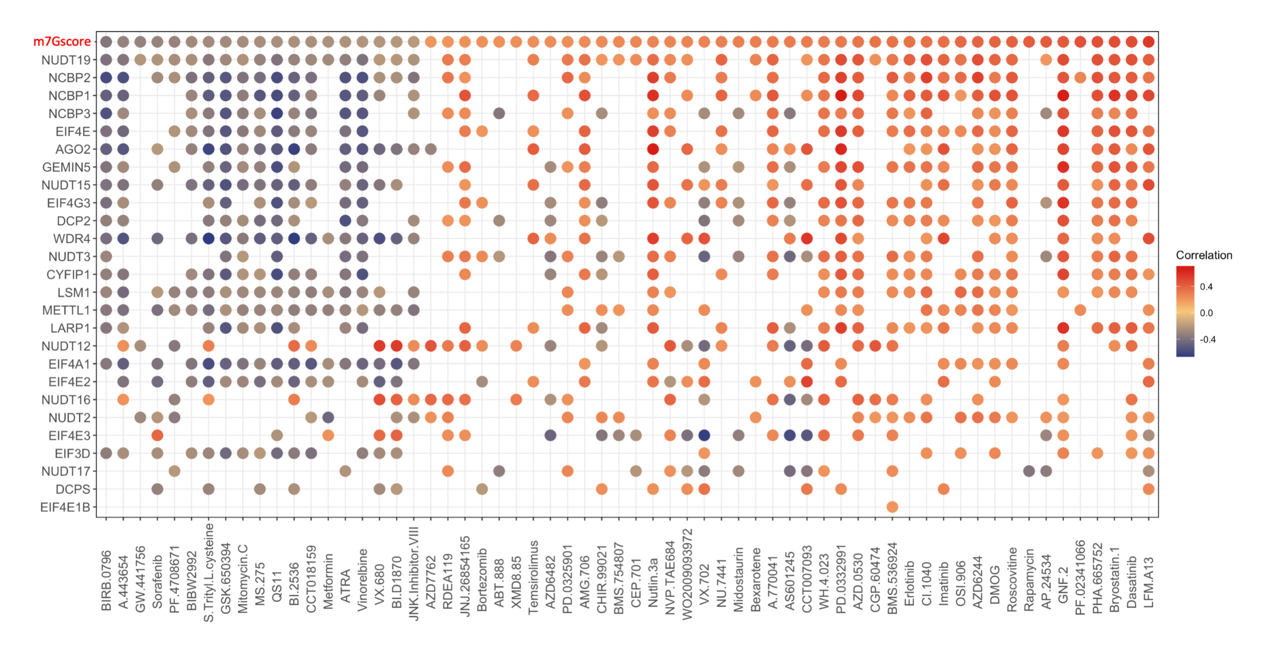


Figure S4. The correlation between the expression of m7G regulators and IC50 of each compound for patients across cancers.
